# Regulation of Riboflavin Biosynthesis in Microorganisms and Construction of the Advanced Overproducers of This Vitamin

**DOI:** 10.3390/ijms26136243

**Published:** 2025-06-28

**Authors:** Justyna Ruchala, Alicja Najdecka, Dominik Wojdyla, Wen Liu, Andriy Sibirny

**Affiliations:** 1 Faculty of Biotechnology, Medical College, University of Rzeszów, Ćwiklińskiej 2D, 35-601 Rzeszów, Poland; awojtun@ur.edu.pl (A.N.); dwojdyla@ur.edu.pl (D.W.); 2 The Doctoral School of the University of Rzeszów, University of Rzeszów, 35-959 Rzeszów, Poland; 3 Department of Molecular Genetics and Biotechnology, Institute of Cell Biology, NAS of Ukraine, Drahomanov Street, 14/16, 79005 Lviv, Ukraine; liuwen12990@gmail.com

**Keywords:** riboflavin production, metabolic engineering, *Bacillus subtilis*, *Ashbya gossypii*, *Candida famata*

## Abstract

Riboflavin (vitamin B_2_) is an essential micronutrient required for all living organisms. It is naturally synthesized by plants and most microorganisms, including the bacterium *Bacillus subtilis*, the filamentous fungus *Ashbya gossypii*, and the yeast *Candida famata*—all of which are known to be riboflavin overproducers. The choice of production organism in industrial applications depends on factors such as yield, ease of cultivation, and the availability of genetic tools. As a result, several microorganisms are commonly used, and their relative prominence can shift over time with advances in metabolic engineering and process optimization. This review presents a comparative analysis of riboflavin biosynthesis across prokaryotic and eukaryotic systems, with a particular focus on regulatory mechanisms governing flavinogenesis. Special attention is given to recent advances in metabolic engineering strategies, including the application of CRISPR/Cas9 genome editing in *Bacillus subtilis* and *Ashbya gossypii*. In yeast systems, significant improvements in riboflavin production have been achieved primarily through the manipulation of transcriptional regulators (e.g., *SEF1*, *SFU1*, *TUP1*) and metabolic genes. The role of other important genes (*PRS3, ADE4, ZWF1, GND1, RFE1, VMA1,* etc.) in riboflavin overproduction in *C. famata* is described. The review also explores the use of alternative, low-cost feedstocks—including lignocellulosic hydrolysates and dairy by-products—to support more sustainable and economically viable riboflavin production. Although considerable progress has been achieved in genetic optimization and bioprocess development, further work is required to fine-tune metabolic flux and maximize riboflavin synthesis, particularly under industrial conditions. This review highlights key opportunities for future research aimed at refining metabolic interventions and expanding the use of renewable substrates for environmentally sustainable riboflavin production.

## 1. Introduction

Riboflavin (vitamin B2) is a water-soluble vitamin that is widely recognized for its essential role in cellular metabolism and is naturally produced by plants, fungi, and most microorganisms. Only protists and some bacteria do not produce riboflavin. Riboflavin is an essential vitamin involved in energy metabolism, oxidative stress response, hormone synthesis, and immune function [1]. The rising awareness of health among consumers and the prevalence of vitamin deficiencies, especially in ageing populations, are driving demand for it. Additionally, research into new applications, such as biofortification and treatments for metabolic disorders [1,2], further boosts potential market growth for riboflavin. The riboflavin market includes feed and food additives, the pharmaceutical, and the cosmetic industry. It should be mentioned that riboflavin used as a feed additive is responsible for approximately 70% of the global riboflavin market [3]. The main global manufacturers, such as Hubei Guangji Pharmaceutical (Wuhan, China), DSM (Maastricht, The Netherlands; since May 2022 DSM-Firmenich), BASF (Ludwigshafen, Germany), Shanghai Acebright Pharmaceuticals (Shanghai, China), and Henan Julong BIO-Engineering Co., Ltd. (Ruzhou City, China), use strains such as *Ashbya gossypii* and *Bacillus subtilis* [3]. The global riboflavin market is estimated to reach USD 508.6 million in 2025. Market growth is expected to continue, with sales projected to rise to USD 872.24 million by 2033, reflecting a compound annual growth rate (CAGR) of 6.98% from 2025 to 2033 [4]. To meet the increasing demand for riboflavin, researchers are focusing on construction of more productive strains, optimizing microbial fermentation processes, and exploring alternative feedstocks.

Riboflavin was first discovered in 1879 from milk whey by Blyth, as a yellow fluorescing substance. Although its isolation, structural description, and nutritional functions took many years to elucidate [5], its significance is now well established. Until the late 20th century, chemical synthesis was the predominant method for riboflavin production. However, microbial fermentation techniques began gaining industrial significance in the 1970s and, by the 1990s, became the primary production method due to their cost-effectiveness and sustainability [3,6]. Riboflavin is chemically synthesized from carbohydrate-based precursors such as D-glucose and D-ribose, typically derived from enzymatic hydrolysis of starch or microbial fermentation [7]. As an alternative to energy-consuming chemical synthesis, microbial fermentation has emerged as a cost-effective and environmentally friendly approach. Microbial fermentation utilizes organisms such as *A. gossypii* or *B. subtilis* to produce riboflavin in a single-step bioprocess, typically using glucose or alternative substrates, including agro-industrial by-products like corn steep liquor and vegetable oils. These alternative feedstocks reduce production costs and contribute to sustainability by repurposing waste materials [5]. Compared to chemical synthesis, microbial fermentation offers significant cost advantages due to lower energy requirements, and the efficient use of renewable substrates throughout the process. While chemical synthesis achieves high purity and consistency, its toxic by-products raise environmental concerns, making fermentation the preferred method in food and pharmaceutical applications. However, challenges remain, particularly in reducing fermentation substrate costs and improving process scalability. Fermentation provides environmental advantages by generating fewer toxic by-products and utilizing renewable resources [7]. A comparison of these two methods of riboflavin synthesis is presented in Table 1.

As previously mentioned, microbial fermentation is currently the primary industrial method for riboflavin production [3,5]. Although microbial fermentation is more sustainable than chemical synthesis, it still faces challenges such as high substrate costs and the need for optimized bioreactor conditions. Many microorganisms synthesize riboflavin, including fungi, yeasts, and most bacteria, but only some can overproduce and excrete it into the culture medium, greatly facilitating subsequent purification. Among them, flavinogenic yeasts are a group of yeast species that overproduce riboflavin under iron deficiency [12,13]. Microorganisms capable of accumulating more than 10 mg/L of riboflavin are considered overproducers. They are further categorized as weak overproducers (approximately 10 mg/L), moderate overproducers (exceeding 600 mg/L), and strong overproducers (over 10 g/L), the latter of which are typically used in industrial production [13]. Genetic modification of the riboflavin biosynthetic pathway enables significantly higher yields through targeted interventions. Advances in genetic engineering have allowed precise modifications of genes involved in riboflavin synthesis, enhancing metabolic pathway efficiency and optimizing production. Key enzymes and regulatory elements can be overexpressed or fine-tuned to increase flux through the pathway, while genes responsible for competing pathways can be knocked out or suppressed to minimize metabolic diversion. The biosynthesis and transport of riboflavin were described in a substantial review by Abbas and Sibirny [14]. The authors of the present review hereby focus on flavinogenic yeast (*Candida famata*), fungus *Ashbya gossypii*, and *Bacillus subtilis*, a widely used bacterial producer, with particular emphasis on genetic engineering strategies for improved riboflavin synthesis and the comparison of these major overproducers.

Among microorganisms with biotechnological potential, *Bacillus subtilis* is definitely worth mentioning due to its numerous advantages, one of the most important of which is the availability of genetic tools that are highly advanced and allow for precise genome editing and metabolic engineering. Numerous approaches for improving riboflavin production in *B. subtilis* have been described [15].

Another notable bacterium is *Corynebacterium ammoniagenes* (formerly *Brevibacterium ammoniagenes*), which has traditionally been employed in the industrial synthesis of nucleotides and nucleosides, including inosine 5′-monophosphate (IMP) and guanosine 5′-monophosphate (GMP). More recently, it has been investigated as a potential riboflavin producer, showing promising results through pathway optimization and gene regulation strategies—achieving titers of 15.3 g/L within 72 h [16].

In parallel, advances in metabolic engineering have established *Escherichia coli* as a competitive platform for riboflavin biosynthesis. Strategies such as overexpression of riboflavin biosynthetic genes, enhancement of precursor pathways, and deletion of regulatory elements—such as FMN riboswitch—have led to titers reaching 21 g/L in fed-batch fermentation [17,18,19,20]. These findings underscore the potential of *E. coli* as a versatile and efficient host for industrial-scale riboflavin synthesis.

To ensure the cost-effectiveness of riboflavin production in these engineered microbial systems, the selection of suitable and economical fermentation substrates remains a critical factor. Because fermentation substrates can be expensive, various waste products—such as agricultural residues, food industry by-products, and crude industrial glycerol—can be metabolized by microorganisms to produce riboflavin. This bioconversion is useful not only for riboflavin production but also for waste recycling and environmental sustainability. This approach to waste utilization helps to reduce the carbon footprint because recycling and reusing materials diminishes reliance on natural resources. Waste utilization through bioconversion technologies not only contributes to the reduction of greenhouse gas emissions but also supports sustainable development and a circular economy. Discussing waste utilization in this review highlights the importance of sustainability and showcases innovative resource management strategies. It also aligns with global efforts to reduce environmental impact, making this review relevant and impactful in addressing contemporary challenges.

## 2. Microbial Biosynthetic Pathways and the Associated Genes Responsible for Production of Riboflavin and Its Precursors

### Overview of Riboflavin Biosynthesis

The earliest research on riboflavin biosynthesis can be traced back to the 1950s [21,22]. Initially, studies focused on filamentous fungi, such as *Eremothecium ashbyii* and *A. gossypii*, as well as yeasts like those of the *Candida* genus [23,24,25]. These organisms were of particular interest due to their natural ability to produce riboflavin at significant levels [14,22].

Later, researchers expanded their studies to include certain eubacteria, such as *Escherichia coli* and *B. subtilis*, as well as non-flavinogenic yeasts like *Saccharomyces cerevisiae*. Investigations into riboflavin synthesis in plants only began on a large scale in the 1990s [14,23].

Riboflavin is a biosynthetic precursor for the coenzymes flavin mononucleotide (FMN) and flavin adenine dinucleotide (FAD), both of which play crucial roles in redox reactions, energy metabolism, and photoreceptor functions [14,26].

The biosynthetic pathway of riboflavin consists of six sequential enzymatic steps, requiring one molecule of GTP and two molecules of ribulose-5-phosphate (Ru5P). These reactions are catalyzed by enzymes encoded by the genes known as *RIB* [22]. The detailed pathway was deciphered largely through the efforts of researchers at the Munich Technical University, Germany (A. Bacher group), alongside contributions from research groups in Cambridge, MA, USA, and Lviv, Ukraine [27].

These foundational studies laid the groundwork for later advances in genetic and metabolic engineering aimed at improving riboflavin yields in microbial hosts.

## 3. Key Genes in Riboflavin Production

### 3.1. Enzymes Catalyzing Riboflavin Biosynthesis

**Figure 1:**
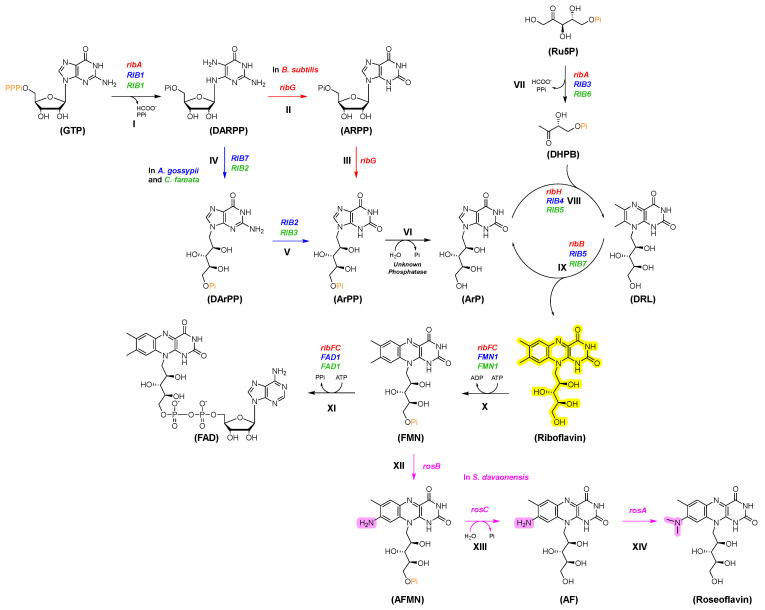
Schematic diagram of the riboflavin and analog pathways in *Bacillus subtilis*, *Ashbya gossypii*, *Candida famata*, and *Streptomyces davaonensis* reproduced from references [14,22,49,62,69]. The enzymatic reaction steps (I–XI) encoded by the genes (*B. subtilis* in red, *A. gossypii* in blue, *C. famata* in green, and *S. davaonensis* in purple) are shown, which are responsible for the conversion of the precursors GTP and ribulose-5-phosphate into riboflavin, FMN, FAD, AF, and roseoflavin. I, GTP cyclohydrolase II; II, 2,5-diamino-6-ribosylamino-4(3H)-pyrimidinone-5′-phosphate deaminase; III, 5-amino-6-ribosylamino-2,4(1H,3H)-pyrimidinedione-5′-phosphate reductase; IV, 2,5-diamino-6-ribosylamino-4(3H)-pyrimidinone-5′-phosphate reductase; V, 2,5-diamino-6-ribitylamino-4(3H)-pyrimidinone-5′-phosphate deaminase; VI, hypothetical phosphatase; VII, 3,4-dihydroxy-2-butanone-4-phosphate synthase; VIII, 6,7-dimethyl-8-ribityllumazine synthase; IX, riboflavin synthase; X, riboflavin kinase; XI, FAD synthetase; XII, AFMN synthase RosB; XIII, novel phosphatase RosC; XIV, dimethyltransferase RosA; GTP, guanosine triphosphate; DARRP, 2,5-diamino-6-ribosylamino-4(3H)-pyrimidinone-5′-phosphate; ARRP, 5-amino-6-ribosylamino-2,4(1H,3H)-pyrimidinedione-5′-phosphate; DArPP, 2,5-diamino-6-ribitylamino-4(3H)-pyrimidinone-5′-phosphate; ArPP, 5-amino-6-ribitylamino-2,4(1H,3H)-pyrimidinedione-5′-phosphate; ArP, 5-amino-6-ribitylamino-2,4(1H,3H)-pyrimidinedione; Ru5P, ribulose-5-phosphate; DHBP, 3,4-dihydroxy-2-butanone-4-phosphate; DRL, 6,7-dimethyl-8-ribityllumazine; FMN, flavin mononucleotide; FAD, flavin adenine dinucleotide; AFMN, 8-demethyl-8-amino-riboflavin-5′-phosphate; AF, 8-demethyl-8-amino-riboflavin.

The riboflavin biosynthetic pathway is initiated by the conversion of GTP, catalyzed by GTP cyclohydrolase II—an enzyme originally isolated from *Escherichia coli* [28]. This enzyme is encoded by the *ribA* gene in *Ashbya gossypii*, and by the *RIB1* gene in *Candida famata* [29,30]. GTP cyclohydrolase II opens the imidazole ring and hydrolytically releases inorganic pyrophosphate from the side chain of the ribose moiety of GTP, resulting in the formation of 2,5-diamino-6-ribosylamino-4(3*H*)-pyrimidinone-5′-phosphate (DARPP) (Figure 1). Studies across multiple organisms, including *B. subtilis*, *Corynebacterium ammoniagenes*, *Helicobacter pylori*, and the flavinogenic yeast *Meyerozyma* (*Pichia*) *guilliermondii*, have provided insights into this enzyme’s properties [14,22].

DARPP is subsequently converted into 5-amino-6-ribitylamino-2,4(1*H*,3*H*)-pyrimidinedione-5′-phosphate (ArPP) through two sequential reactions: reduction and deamination (Figure 1). In eubacteria, DARPP is first deaminated to 5-amino-6-ribosylamino-2,4(1*H*,3*H*)-pyrimidinedione-5′-phosphate (ARPP), and the ribose moiety of the intermediate ARPP is then reductively ring-opened to generate ArPP, which is catalyzed by a bifunctional enzyme (gene *ribG* in *B. subtilis*) [31,32].

However, the order of these two reactions is reversed in archaea and fungi, including yeasts: the ribosyl side chain of DARPP is first reduced to 2,5-diamino-5-ribitylamino-4(3*H*)-pyrimidinone-5′-phosphate (DArPP) catalyzed by DARPP reductase (*RIB7* in *A. gossypii*, *RIB2* in *C. famata*). Then, the DArPP is deaminated by the enzyme DArPP deaminase (*RIB2* in *A. gossypii*, *RIB3* in *C. famata*) with the formation of ArPP [33,34] (Figure 1).

ArPP undergoes further dephosphorylated into 5-amino-6-ribitylamino-2,4(1*H*,3*H*)-pyrimidinedione (ArP) by the catalytic action of a non-identified yet specific phosphatase or a non-specific phosphatase, which means that the mechanism of dephosphorylation is still unknown [14,23].

The other initial precursor of riboflavin biosynthesis, Ru5P, is transformed into 3,4-dihydroxy-2-butanone-4-phosphate (DHBP) through a skeletal rearrangement catalyzed by the DHBP synthase (*ribA* in *B. subtilis*, *RIB3* in *A. gossypii*, and *RIB6* in *C. famata*) [29,35,36,37] (Figure 1). This reaction is characterized by extraordinary complexity, and the DHBP synthase has now been isolated and identified from many microorganisms [35,38,39,40,41].

In the next step, the two different branches merge into one. ArP condenses with DHBP to form 6,7-dimethyl-8-ribityllymazine (DRL) catalyzed by DRL synthase (or lumazine synthase, *ribH* in *B. subtilis*, *RIB4* in *A. gossypii*, and *RIB5* in *C. famata*) [33,34,42,43] (Figure 1), which was first isolated from *B. subtilis* as a complex with riboflavin synthase [44].

In the final step, two molecules of DRL are transformed into one riboflavin and one precursor molecule, ArPP, via an unusual dismutation, which is catalyzed by the riboflavin synthase (*ribB* in *B. Subtilis*, *RIB5* in *A. gossypii*, and *RIB7* in *C. famata*) [30,44,45,46] (Figure 1).

FMN, also known as riboflavin-5′-phosphate, is formed by phosphorylation of riboflavin in the C-5′-position of the ribityl chain catalyzed by riboflavin kinase (*ribFC* in *B. subtilis*, *FMN1* in *A. gossypii* and *C. famata*) [47,48,49,50]. In total, two groups of riboflavin kinases have been identified. One group is represented by monofunctional riboflavin kinase proteins in fungi, plants, archaea, and eubacteria (rarely) [51,52,53,54,55]. The other group is bifunctional riboflavin kinase/FAD synthetase, which has been found to be the basic enzyme in eubacteria [47,56]. FAD synthetase (*ribFC* in *B. subtilis*, *FAD1* in *A. gossypii* and *C. famata*) catalyzes the transfer of adenylyl residues from ATP to FMN and generates FAD [49,56,57,58] (Figure 1).

Roseoflavin (8-demethyl-8-dimethylamino-riboflavin) is a natural riboflavin analog with antibiotic activity, produced by the bacteria *Streptomyces davaonensis* and *Streptomyces cinnabarinus* [59,60]. Its biosynthesis starts from flavin mononucleotide (FMN) and is catalyzed by the enzyme 8-demethyl-8-amino-riboflavin-5′-phosphate (AFMN or AFP) synthase, encoded by the *rosB* gene, resulting in the formation of AFMN. [61]. Then, AFMN is subsequently dephosphorylated to 8-demethyl-8-amino-riboflavin (AF) by a specific phosphatase *RosC* [62,63]. Finally, AF is converted to roseoflavin by the dimethyltransferase *RosA* [64] (Figure 1).

### 3.2. Metabolic and Genetic Regulation of Riboflavin Biosynthesis

The regulation of riboflavin biosynthesis differs significantly among microbial species. In prokaryotes, a key regulatory mechanism involves FMN riboswitches—structured RNA elements typically located in the 5′ untranslated region of mRNAs. These riboswitches bind flavin mononucleotide (FMN), a direct product of riboflavin metabolism, and act as sensors to modulate gene expression. When intracellular FMN levels are high, the riboswitch undergoes a conformational change upon FMN binding, which can lead to transcription termination or inhibition of translation initiation, thereby downregulating genes involved in riboflavin biosynthesis. This mechanism is well characterized in *B. subtilis* [14,65].

In *C. famata*, riboflavin biosynthesis is tightly regulated by iron availability via the Sef1 transcription factor, which activates *RIB* gene expression under iron-limiting conditions [66]. In contrast, *A. gossypii* does not respond to iron levels but instead links riboflavin overproduction to sporulation and oxidative stress. The primary regulatory mechanism in *A. gossypii* involves the interplay of histone deacetylases (e.g., HST1 and HST3), which modulate chromatin accessibility at riboflavin biosynthetic *loci* [67]. Key transcriptional regulators include *SEF1*, which enhances riboflavin production by upregulating *RIB* gene expression, and *TUP1*, a repressor that modulates gene expression in response to iron availability [66,68].

The regulatory mechanisms governing riboflavin biosynthesis are highly diverse and species-specific, presenting both challenges and opportunities for metabolic engineering. A comprehensive understanding of these distinct regulatory networks serves as a crucial foundation for the rational design of targeted strategies aimed at enhancing riboflavin production across various industrial microbial hosts. Below, the specific regulatory mechanisms involved in the major riboflavin producers are described.

## 4. *Bacillus subtilis*

Wild-type *B. subtilis* produces riboflavin at low levels; however, through metabolic engineering and mutagenesis, industrial strains can be isolated which achieve high production yields. During the past two decades, it has become the main producer of riboflavin [70].

The European Food Safety Authority (EFSA) Panel on Additives and Products or Substances used in Animal Feed (FEEDAP) concluded that the use of riboflavin produced by *B. subtilis* KCCM 10445 [71] and *B. subtilis* CGMCC 13326 [72] was safe for the target species, the consumer, and the environment. Additionally, through a comprehensive characterization of the enzymes involved in the riboflavin biosynthetic pathway, *B. subtilis* has been successfully engineered to serve as a cell factory for riboflavin production [47,73,74]. Therefore, *B. subtilis* has been the species of choice for commercial riboflavin production [3,15,74].

Many studies have focused on precursor supply and regulation of the key enzymes, which were considered to be the two major limiting factors in riboflavin production [38,75].

In *B. subtilis*, Ru5P and GTP are precursors in the riboflavin biosynthesis pathway. The main generation of Ru5P in *B. subtilis* occurs in the pentose phosphate pathway (PPP) with an additional role for the gluconate pathway.

In the oxidative branch of PPP, glucose-6-phosphate (G6P) is first converted to 6-phosphogluconolactone by G6P dehydrogenase and then hydrolyzed by lactonase to 6-phosphogluconic acid, which is further decarboxylated to Ru5P by 6-phosphogluconate dehydrogenase. In the non-oxidative pentose phosphate pathway, fructose-6-phosphate and glyceraldehyde-3-phosphate undergo epimerase, isomerase, transaldolase, and transketolase reactions involved in interconversions of phosphorylated sugars [76,77,78]. In the gluconate pathway, glucose is oxidized by glucose dehydrogenase to gluconate, which is then phosphorylated by gluconate kinase to 6-phosphogluconate and further decarboxylated to produce Ru5P [79,80,81].

Several metabolic engineering strategies have been developed to modify the flux of the synthesis pathways and increase the availability of Ru5P in *B. subtilis* strains. To enhance the intracellular pool of Ru5P, the *ZWF* gene under the control of the inducible Pxyl promoter was overexpressed in *B. subtilis* PY, leading to 25% increased riboflavin production [82]. The individual overexpression of the *zwf* and *gnd* genes from *Corynebacterium glutamicum* in *B. subtilis* RH33 led to approximately 18% and 22% increased riboflavin production, respectively; moreover, co-expression of the two genes led to a 31% increase in riboflavin production [83]. In the first step of the non-oxidative branch of the PPP, ribulose-5-phosphate 3-epimerase (encoded by the *rpe* gene) converts Ru5P into xylulose 5-phosphate. Yang et al. constructed the strain with inactivation of Rpe in *B. subtilis* LY, resulting in a five-fold increase of riboflavin production compared to the parental strain [84].

**Figure 2:**
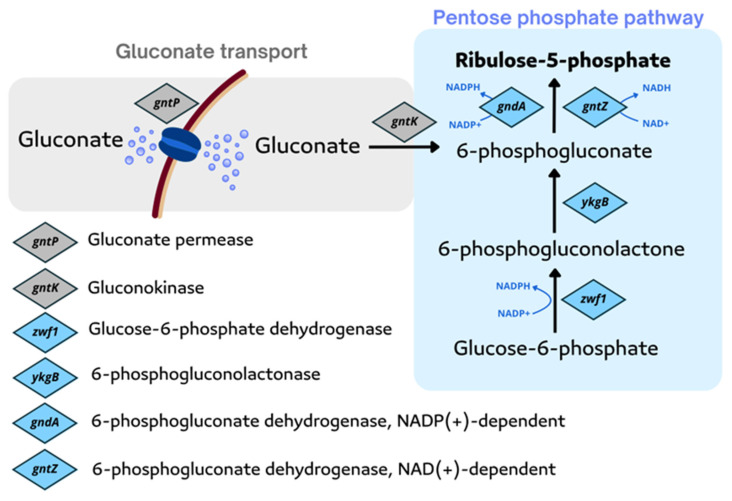
Metabolic engineering strategies targeting the pentose phosphate pathway (PPP) in *Bacillus subtilis* to enhance riboflavin production, including carbon flux redirection via the gluconate pathway.

Additionally, Zhang et al. overexpressed the *gntP* (encoding gluconate permease), *gntK* (encoding gluconokinase), and *gntZ* (encoding phosphogluconate dehydrogense) genes in the gluconate pathway to redirect the carbon flux into the PPP; moreover, using sodium gluconate, which is a cheap bulk material, instead of glucose, resulted in a 56.3% increase in riboflavin titer [85] (Figure 2).

A deeper understanding of central carbon metabolism is essential for designing effective metabolic engineering strategies aimed at enhancing riboflavin production [14].

Gluconeogenesis is a biological process leading to the generation of glucose from non-sugar carbon substrates in central carbon metabolism. Its modification has been successfully applied to redirect the carbon flux toward the PPP in *C. glutamicum* [86,87]. Additionally, it also has been suggested that further improvement of riboflavin production by deregulating the key enzymes involved in gluconeogenesis could be feasible [75].

Wang et al. investigated the effects of deregulation of gluconeogenesis on the improvement of riboflavin production by overexpression of the critical gluconeogenic genes in strain *B. subtilis* RH33. The resulting data showed that co-overexpression of the *gapB* (encodes NADPH-dependent glyceraldehyde 3-phosphate dehydrogenase) and *fbp* (encodes fructose-1,6-biphosphatase) genes led to a 21.9% increase in riboflavin titers up to 4.89 g/L in shake-flask fermentation. The study demonstrated that deregulation of gluconeogenesis is an effective strategy to enhance riboflavin production, as well as other metabolites directly from PPP- and NADPH-dependent compounds using glucose as a carbon source [73]. GTP is produced in cells through the de novo purine synthesis pathway, which consists of ten different enzymatic reactions to generate inosine-5′-monophosphate (IMP) from 5-phosphoribosyl-1-pyrophosphate (PRPP) [88]. Next, IMP is converted to GTP and ATP.

Early studies have shown that *B. subtilis* mutants resistant to 8-azaguanine (purine analog), DL-methionine sulfoxide (glutamine antagonist), or decoyinine (purine synthesis inhibitor) increased GTP generation and riboflavin production due to deregulation in the purine pathway [89,90,91,92].

An operon is a group of genes regulated by a single promoter and transcribed as single policistronic mRNA. It is common in prokaryotes; nevertheless, numerous examples now exist of eukaryotic gene clusters composed of functionally related yet non-homologous genes, forming functional gene arrangements that share some operon-like characteristics, such as physical grouping and coordinated regulation but not the policistronic mRNA [93]. Genes involved in the purine pathway are clustered as *pur* operon (*purEKBCSQLFMNHD*) in *B. subtilis*. The transcription of all genes starts with a *δ*A-type promoter upstream of the *purE* gene, in which no internal promoter has yet been identified [94]. Recent studies have focused on genetically engineering targeted modifications of purine operon regulatory elements to increase gene expression levels of the purine pathway. Expression of *pur* operon is subject to two types of regulation: transcriptional initiation and transcriptional weakening [95]. Special DNA sequences called *pur*-Boxes are present in the upstream control region of the purine operon transcription initiation site [96].

The PurR-*Pur*Box system is involved in purine synthesis, transport, and metabolic function [97]. When cells contain high concentrations of PRPP, which binds to the repressor protein PurR (encoded by the *purR* gene), preventing PurR from binding to *pur*-Box and thereby allowing normal transcription of the purine operon [96,98]. Besides regulating the expression of purine pathway genes, PRPP also competes with adenosine-5′-monophosphate (AMP) for the catalytic binding site of PRPP amidotransferase, encoded by *purF*, which is a key regulatory enzyme in the de novo purine biosynthesis pathway [99].

Knock out of the *purR* gene in *B. subtilis* 168 appeared to be an effective strategy for riboflavin overproduction [100]. Similar results were obtained in other work on deletion of the *purR* gene [88]. The data showed that the metabolic flux through the purine biosynthesis pathway could be successfully improved, resulting in a maximum riboflavin production of 827 mg/L in shake-flask fermentation by the engineered strain BS110 [88].

GTP can also be synthesized from purine bases or purine nucleosides catalyzed by purine phosphoribosyl transferases or nucleoside kinases, respectively, in the purine salvage pathway. Knock out of adenine phosphoribosyl transferase (encoded by the *apt* gene), xanthine phosphoribosyl transferase (encoded by the *xpt* gene), and adenine deaminase (encoded by the *adeC* gene) increased riboflavin production by 14.02%, 6.78%, and 41.50% in the background of strain BSRP (*purRΔ*), respectively [101]. These results were apparently obtained due to a drop in production of adenine nucleotides and a consequent increase in GTP accumulation.

**Figure 3:**
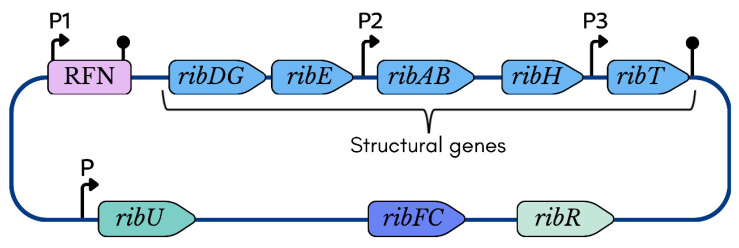
Scheme of *rib* operon in riboflavin biosynthesis regulation by *B. subtilis* reproduced from references [3,7]. RFN, chromosomal FMN-specific element; *ribDG-E-AB-H-T*, *rib* operon; *ribU*, gene encoding riboflavin transporter; *ribFC*, gene encoding bifunctional flavokinase/FAD synthetase; *ribR*, gene encoding monofunctional flavokinase RibR; P1, P2, and P3 denote confirmed promoters (indicated by arrows); P, predicted promoter (indicated by arrows). The hairpin symbols denote confirmed transcription terminators.

In *B. subtilis*, the total length of *rib* operon is nearly 4.3 kb, and it consists of five genes: *ribG*, *ribB*, *ribA*, *ribH*, and *ribT* (Figure 3). Among them, *ribA* and *ribG* encode bifunctional (2-domain-containing) enzymes.

In contrast to fungi, the regulation of riboflavin biosynthesis regulation in *B. subtilis* is controlled by feedback repression of the *rib* operon through the riboswitch FMN-specific element (RFN) [102,103,104] (Figure 3). This highly conserved RNA motif selectively binds to the coenzyme FMN and regulates the expression of FMN biosynthesis-associated genes [104,105]. The *rib* operons have also been studied in *Bacillus amyloliquefaciens*, *Bacillus halodurans*, *Bacillus abortus*, *Shewanella oneidensis*, and *C. glutamicum*, etc. [65,106].

The *ribA* gene encodes GTP cyclohydrolase II at its 3′-end and DHBP synthase at its 5′-end, which react with the precursors GTP and Ru5P, respectively [22]. Overexpression of a truncated *ribA* gene decreased riboflavin production in *B. subtilis* RB50::[pRF69]_n_::[pRF93]_m_ Ade^+^, while introduction of the intact *ribA* gene increased riboflavin production by 25% [38]. Hohmann et al. inserted the strong *VegI* promoter into the chromosome that drives the expression of the *ribA* gene, which led to an increase in riboflavin yield [107].

In addition, genetic manipulation of the *rib* operon was performed in *B. subtilis* strain BS77, including overexpression of the *ribA* gene, using the strong promoter *P43*, and deletion of the *ribO* gene, which functions to regulate the transcription of riboflavin biosynthesis pathway genes. As a result, the constructed *B. subtilis* strain BS89 was 1.4-fold higher than that of parental strain [88].

The *ribG* gene encodes bifunctional DARPP deaminase and ARPP reductase activity, with a deaminase encoded at the 5′-end and a reductase at the 3′-end [32] (Figure 3). Both genes *ribB* and *ribH* encode riboflavin synthase, which is a complex enzyme consisting of a light enzyme (3 α-subunits) and a heavy enzyme (approximately 60 β-subunits) [22]. The *ribH* gene encodes the β-subunits of riboflavin synthase, also known as lumazine synthase, which catalyzes the reaction between the 5-position amino group of ArP and the carbonyl group of DHBP to eventually produce DRL [14,22]. The *ribB* gene encodes the α-subunits of riboflavin synthase that catalyze the dismutation of two molecules of DRL, the immediate precursor of riboflavin synthesis, yielding one molecule of riboflavin and one molecule of ArPP [14,22].

Recently, CRISPR-based genomic editing has been developed for multiplex gene editing in *B. subtilis* [108,109]. CRISPR-Cas9n (Cas9 nickase) is a modified version of CRISPR-Cas9 that generates single-strand nicks, reducing off-target effects and thereby enhancing genome editing precision. The improved CRISPR-Cas9n-mediated multiplexing system reached an efficiency of 65% for three-point mutations in genes *ribA*, *ribB*, and *ribH* (Figure 3) [108].

The *ribT* gene is located at the end of this operon and its function had not been elucidated (Figure 3) [110], until recent research revealed that its enzyme belongs to a member of GCN5-related N-acetyltransferase, which transfers the acetyl group from acetyl-CoA to a variety of substrates [111].

The *ribFC* and *ribR* genes play an indirect regulatory role in *rib* operon expression (Figure 3), [112]. The *ribFC* gene of bifunctional flavokinase/FAD-synthetase is not linked to the riboflavin operon, but is located elsewhere in the chromosome [104]. Moreover, a *B. subtilis* mutant of the *ribFC* gene led to an increase in riboflavin concentration up to 15 g/L [113].

The *ribR* gene encodes an RNA-binding protein that is also not part of the *rib* operon (Figure 3), and is believed to act as a regulatory protein as it seems to intervene with the RFN function [104,114].

Furthermore, the introduction of the *ribM* gene (encoding the energy-independent functional riboflavin transporter RibM) from *S. davaonensis* into a high-performance *B. subtilis* riboflavin production strain increased riboflavin export [115]. It was the first example suggesting that the improvement of riboflavin excretion is a useful strategy to increase the riboflavin yield in *B. subtilis* similarly to the case of *C. famata* yeast.

Most efforts have focused on engineering the regulation of the *B. subtilis rib* operon and on overexpressing its structural genes *ribGBAHT* [14,15] (Figure 3).

Highly efficient riboflavin-producing strains were developed by introducing additional copies of the *ribGBAHT* genes (Figure 3), which were regulated by either strong native promoters or strength-optimized synthetic bacterial and phage promoters [116,117]. However, the industrial production of riboflavin in *B. subtilis* still faces several unresolved challenges, including the *ribR*-mediated regulation of FMN riboswitches, which limits riboflavin production; the unidentified phosphatases involved in the riboflavin biosynthesis pathway; the reactivity of flavins, which can damage cells; and the absence of an efficient transport system for actively exporting flavins, unlike *A. gossypii* [118].

In addition to the method reviewed above, optimizing cultivation conditions is also a viable approach to increasing riboflavin production in *B. subtilis* [57,119].

Oxygen dissolved in the culture medium is one of the most important factors in the fermentation process and is related to cell biomass accumulation and riboflavin production [120,121,122].

Man et al. investigated the effects of the change of agitation speed on riboflavin production by engineered *B. subtilis* RF1 in fed-batch fermentation. A strategy of gradually increasing the agitation speed from 600 to 900 r/min was established and led to a 21.4% increase in riboflavin yield compared to using a single speed of 600 r/min [123].

Oraei et al. selected three minerals, FeSO_4_, MgSO_4_, and K_2_HPO_4_, as the supplements of the medium, which significantly affected riboflavin production, from thirteen different minerals via the Plackett–Burman design. Subsequently, an optimized medium containing fructose, yeast extract, FeSO_4_, MgSO_4_, and K_2_HPO_4_ at concentrations of 38.10, 4.37, 0.02, 0.85, and 2.27 g/L, respectively, resulted in a riboflavin titer of 11.73 g/L after 72-h shake-flask fermentation. [124].

Along with these external factors, the regulation of riboflavin biosynthesis itself plays a crucial role in optimizing production. The FMN riboswitch is an RNA regulatory element located in the 5′ untranslated region (5′ UTR) of mRNAs involved in riboflavin biosynthesis. It consists of two key regions: the aptamer domain, which specifically binds FMN, and the expression platform, which determines the regulatory outcome. The aptamer domain features a conserved three-dimensional structure with helices, loops, and a binding pocket that accommodates FMN through hydrogen bonding, π-stacking, and van der Waals interactions [125,126,127].

Upon FMN binding, the riboswitch undergoes a conformational change that affects gene expression. At the transcriptional level, FMN binding induces the formation of a transcription terminator hairpin, halting RNA polymerase and preventing transcription. At the translational level, FMN binding sequesters the Shine–Dalgarno sequence within a stem-loop, blocking ribosome binding and translation. Additionally, FMN binding can destabilize the RNA, promoting degradation and further reducing gene expression. This ligand-specific, energy-efficient mechanism provides a feedback loop where elevated FMN levels directly repress riboflavin biosynthesis by regulating transcription, translation, or RNA stability without the need for protein factors [127,128].

## 5. *Ashbya gossypii* (*Eremothecium gossypii*)

**Figure 4:**
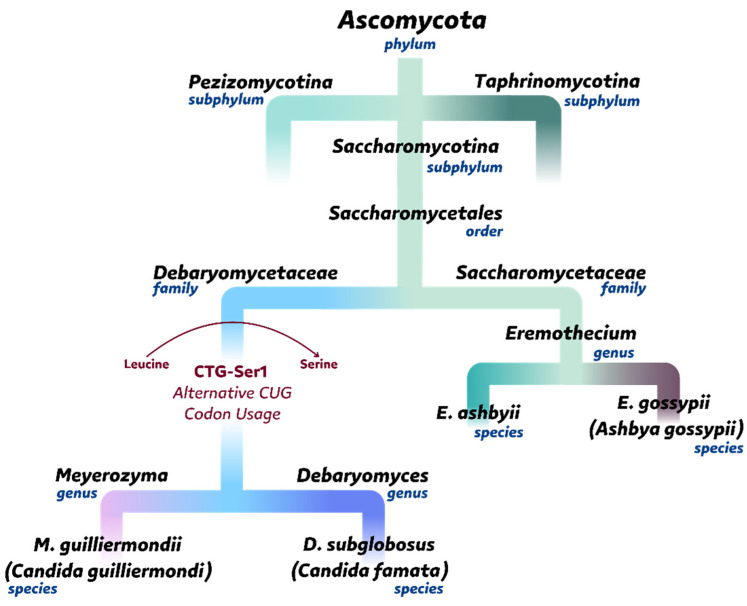
The schematic provides a detailed representation of the relationships between the filamentous fungus *A. gossypii* and the yeasts *C. famata* and *C. guilliermondii*, emphasizing their phylogenetic connections. The diagram also highlights the alternative names commonly used for these species, reflecting their interchangeable usage in various contexts. Additionally, a notable feature included in the graphic is the alternative CTG-Ser1 codon usage, a unique characteristic of the mentioned yeasts, which distinguishes them from standard genetic code usage. This visual summary offers valuable context for understanding both their evolutionary relationships and specific genetic traits.

*A. gossypii* is one of the earliest known riboflavin producers with industrial potential, first described by Guillermond in the early 20th century. Its taxonomic classification has evolved over time, but genome sequencing ultimately placed it within the order *Saccharomycetales* (Figure 4). *A. gossypii* and *Eremothecium ashbyii* are closely related filamentous fungi, both capable of overproducing riboflavin. *A. gossypii* is the preferred organism for industrial riboflavin production and has been more extensively studied than *E. ashbyii.* Although *E. ashbyii* has similar biosynthetic capabilities, its genetic instability limits its use in industrial applications [14,129,130]. Notably, *E. ashbyii* can also oversynthesize FAD and secrete it into the culture medium [131].

The fermentation process of *A. gossypii* consists of three main stages: (1) exponential growth phase, (2) stationary phase with riboflavin accumulation, and (3) sporulation phase, during which riboflavin is released into the medium. Riboflavin is produced after a few days of incubation when hyphal tips from mycelia are converted to sickle-shaped spores [132]. *A. gossypii* is able to grow on divergent media composed of carbohydrates, glycerol, and yeast extract, which promotes exponential growth, or vegetable oils which favor riboflavin synthesis [133,134]. It has been found that nutritional stress, which causes a decline in growth rate, as well as oxidative stress, is associated with riboflavin overproduction [34,135].

Initially, researchers analyzed flavinogenesis by supplementing different substances to the culture of *A. gossypii*, identifying those with a positive influence (ribitol, glycine) [136] or a negative influence (8-azaadenine, 8-hydroxyquinoline) on flavinogenesis [137]. Further research on riboflavin synthesis focuses on optimizing fermentation conditions [138,139]. Since the riboflavin production pathways have been established and genome sequencing has been completed [140], molecular cloning tools have enabled gene manipulations. The first genetic modifications were made by the BASF company. This approach resulted not only in a higher riboflavin yield but also in the production of recombinant proteins, microbial lipids (Single Cell Oil—SCO), flavor compounds, and nucleosides [45,141].

To date, *A. gossypii* has been utilized in industrial riboflavin production for over twenty years (Schwechheimer et al., 2016 [5]; Liu et al., 2023 [15];). Current studies primarily focus on the molecular level [67,135].

**Figure 5:**
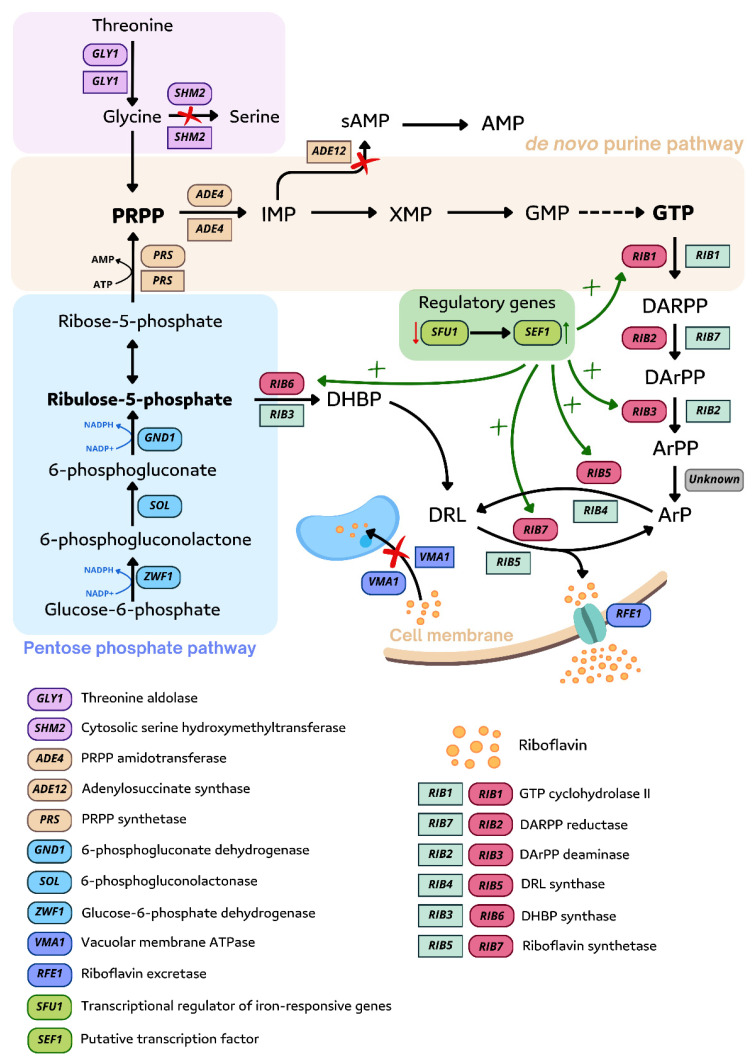
The scheme illustrates the key metabolic modifications that have enhanced riboflavin production efficiency in *C. famata* and *A. gossypii*. Pathways and modifications are color-coded for clarity: the pentose phosphate pathway is highlighted in blue, alterations in glycine synthesis are shown in purple, and the de novo purine synthesis pathway is marked in beige. All genes written in an oval shape refer to modifications carried out in the yeast *C. famata*, while those shown in a rectangle refer to modifications in *A. gossypii.* A red marker indicates modifications intended to inhibit the pathway. The diagram also highlights the riboflavin biosynthetic pathway, detailing each step along with the corresponding genes (*RIB*) involved in *C. famata* and *A. gossypii*. This comprehensive representation allows for a clear comparison of the genetic and enzymatic contributions at each step of the pathway in these two species. The green arrow indicates activation by the product of the *SEF1* gene, the structural gene *RIB1* involved in riboflavin biosynthesis. Similarly, the *SEF1* product activates the structural genes *RIB3, RIB5, RIB6*, and *RIB7* but not *RIB2* (S. Romanov, O. Lyzak, A. Sibirny, K. Dmytruk, unpublished).

Since all the *RIB* genes have been identified, efforts have been made to improve riboflavin production by overexpressing these genes. It has been shown that the overexpression of five *RIB* genes in *A. gossypii* (*RIB1, RIB2, RIB3*, *RIB5*, and *RIB7*) increases riboflavin production 3.1-fold (326.6 mg/L). Blocking the conversion of inosine 5′ monophosphate to adenosine monophosphate by deleting the *ADE12* gene resulted in a 2.5-fold increase in riboflavin synthesis (246 mg/L) (Figure 5). Simultaneous overexpression of the mentioned *RIB* genes and inactivation of the *ADE12* gene further increased riboflavin by 5.4-fold (523 mg/L) compared to the wild-type strain [102].

In addition to genes directly involved in the riboflavin biosynthesis pathway, researchers have also focused their efforts on enhancing the production of key precursor guanosine triphosphate (GTP). To achieve higher yields of GTP, genetic manipulations of the purine biosynthesis pathway, which governs the production of GTP, have been implemented (Figure 5).

As previously mentioned, PRPP is a molecule associated with riboflavin synthesis. In *A. gossypii*, *PRS* genes encoding PRPP synthetase are subject to feedback inhibition by ADP. The *A. gossypii* genes *AGR371C* and *AGL080C* have orthologs in *S. cerevisiae* (*PRS2,4* and *PRS3* respectively). Specific amino acid substitutions eliminate ADP inhibition: leucines located at positions 133 in *PRS2,4* and 132 in *PRS3* were replaced with isoleucine, while histidines at positions 196 in *PRS2,4* and 195 in *PRS3* were substituted with glutamine. Overexpression of *PRS* genes, with simultaneous elimination of ADP inhibition, significantly increased riboflavin production yield by 80% compared to the wild-type strain [142].

Additionally, overexpression of the *ADE4* gene which encodes PRPP amidotransferase along with modifications to prevent inhibition by purine triphosphate derivatives, improved riboflavin synthesis by 10-fold (228 mg/L) [103]. This strategy not only demonstrates the critical role of purine metabolism in riboflavin biosynthesis but also highlights the potential of genetic modifications to optimize metabolic pathways for enhanced industrial production of riboflavin.

As was noted earlier, glycine has a positive impact on riboflavin biosynthesis. In 1998, Monschau et al. overexpressed the *GLY1* gene encoding threonine aldolase which is responsible for glycine synthesis from threonine under control of the *TEF* promoter and terminator. This resulted in increased riboflavin production by nine-fold but only with additional threonine supplementation [143]. Alternatively, *SHM* genes encode serine hydroxymethyltransferase, which is responsible for transformation of glycine to serine. This reaction reduces the availability of glycine, which is necessary for the formation of the key precursor of purine nucleotides—PRPP, so it seemed advisable to attempt to inhibit this process. In *A. gossypii*, two of the mentioned genes have been identified: *SHM1* (mitochondrial) and *SHM2* (cytosolic). Disruption in the *SHM2* gene led to a higher glycine supply, which increased riboflavin biosynthesis by 10-fold [144] (Figure 5).

Another study focused on riboflavin transport. Disruption of the *VMA1* gene, which is responsible for accumulating riboflavin in the vacuole compartment, resulted in the complete excretion of riboflavin into the medium and thus elevated total riboflavin accumulation [145].

Riboflavin plays a crucial role in metabolic and redox processes as a precursor to FAD and FMN, essential cofactors for enzymes involved in oxidative phosphorylation and energy metabolism. FMN and FAD are coenzymes mainly involved in key redox reactions that contribute to NADH generation, primarily in the electron transport chain (Complex I), the Krebs cycle (alpha-ketoglutarate dehydrogenase, succinate dehydrogenase), and beta-oxidation of fatty acids. While FMN directly oxidizes NADH in Complex I, FAD participates in reactions like succinate dehydrogenase (Krebs cycle) and beta-oxidation (cofactor for fatty acyl-coA dehydrogenases), indirectly influencing NADH production (Adeva-Andany et al., 2019 [146]; Hirst, 2013 [147]; Robinson & Lemire, 1996 [148]; Rutter et al., 2010 [149]).

These processes generate NADH, which maintains physiological NAD^+^ levels, a critical molecule for sirtuin activity. Sirtuins, being NAD^+^-dependent, regulate key cellular functions related to energy metabolism, mitochondrial health, oxidative stress response, and aging. Thus, riboflavin indirectly supports sirtuin functions by maintaining the cellular redox balance and energy metabolism. *A. gossypii* possesses four sirtuin genes: *HST1, HST2, HST3*, and *HST4*. Kato et al. identified that disruption in two of these genes, AgHST1 and AgHST3, regulates riboflavin production in *A. gossypii* through two mechanisms: modulating histone H3 acetylation and enhancing NAD biosynthesis, leading to a 4.3- and 2.9-fold increase in riboflavin production, respectively [67].

Choosing an appropriate promoter is critical in molecular cloning because it controls the expression of the inserted gene. There are several strong native constitutive promoters commonly used in *A. gossypii* (pGPD1, pTEF, pPGK1, and pADH1), but, recently, new ones have been discovered (pCCW12, pSED1, pTSA1, and others) with potential applications for metabolic engineering [150]. Moreover, the CRISPR/Cas9 system has gained popularity in genome editing and has been successfully used in *A. gossypii*, for example, to improve the uptake of xylose [151,152]. Additionally, disparity mutagenesis has proven useful for creating new *A. gossypii* mutants for future gene manipulation. This method deliberately introduces an imbalance in the replication fidelity of the two DNA strands. It was proposed by Furusawa and Doi in 1998 as a mechanism to accelerate evolution. During DNA replication, each cell synthesizes two new strands—the leading strand and the lagging strand. Under normal conditions, both strands are copied with high accuracy, minimizing errors. However, in disparity mutagenesis, this process is disrupted—the leading strand is synthesized with high fidelity (almost no mutations), while the lagging strand is replicated with lower accuracy, leading to an accumulation of mutations. As a result, greater genetic diversity appears among the offspring cells, since some retain the original genetic material without mutations, while others accumulate changes that may lead to beneficial traits [153]. This mechanism is particularly useful in genetic engineering because it allows for the rapid generation of mutants with desirable characteristics, such as increased riboflavin production in *A. gossypii.* By producing mutants with altered phenotypes, disparity mutagenesis provides a foundation for exploring gene functions, optimizing metabolic pathways, and engineering strains with improved production capacities [154].

## 6. *Meyerozyma* (*Candida*, *Pichia*) *guilliermondii*

*Meyerozyma (Pichia) guilliermondii* was first described in 1945 by Tanner and co-authors as a non-conventional yeast capable of overproducing riboflavin [12]. Previously, in 1912, it was named *Endomyces guilliermondii* by Castellani [155]. In 2010, it was officially renamed *M. guilliermondii* (Kurtzman & Suzuki, 2010 [156]) (Figure 4).

It is a strictly aerobic microorganism with an optimal growth temperature of 30 °C (up to 42 °C). *M. guilliermondii* is frequently considered a model organism for flavinogenic yeasts because it is one of the few species in which both classical and molecular genetic methods have been established. Moreover, it has been used to study the enzymology of riboflavin synthesis, as it overproduces the enzymes and intermediates involved, as well as the systems responsible for the active transport of the riboflavin into and out of the cell. Additionally, it has been instrumental in identifying genes that play either a positive or negative role in this regulatory process [157].

It is the only reported microorganism capable of actively transporting riboflavin and accumulating it within a cell [158,159]. Some strains of *M. guilliermondii* are also capable of efficiently converting xylose to xylitol [160].

One of the first conclusions during research was focused on the impact of iron on riboflavin production. Iron limitation enhances riboflavin synthesis primarily through activation of the *SEF1* transcription factor, which regulates *RIB1* and plays a role in flavinogenesis [161]. Later, it was discovered that iron represses the activity of all enzymes involved in riboflavin synthesis, except for the one catalyzing the second step [162]. Additionally, iron limitation reduces the activity of catalase and superoxide dismutase (Prokopiv, 2013 [163]). Moreover, mutations in specific genes (*RIB80, RIB81*, and *HIT1*) positively affect both riboflavin production and iron accumulation (Boretsky et al., 2007 [164]; Fedorovich et al., 1999 [165]).

The structural genes responsible for riboflavin synthesis—*RIB1* (GTP cyclohydrolase II), *RIB2* (DARPP reductase), *RIB3* (DARPP deaminase), *RIB4* (function not fully characterized), *RIB5* (lumazine synthase), *RIB6* (DHBP synthase), and *RIB7* (riboflavin synthase) from *D. hansenii*—were cloned into riboflavin-deficient mutants of *M. guilliermondii,* successfully restoring their riboflavin biosynthesis [33].

Oxidative stress has a significant influence on riboflavin production, especially when combined with iron accumulation. It has been shown that cells treated with a superoxide-generating agent (methylviologen) exhibit increased production of riboflavin [166].

Another study focused on *YFH1* gene, which is involved in iron transport and accumulation. Deletion of this gene, which encodes yeast frataxin homologue, caused multiple effects on metabolism. It disrupted the sulfate assimilation pathway and led to changes in superoxide dismutase activities [167].

Along with riboflavin, gluthatione (GSH) plays a key role in protecting cells from oxidative damage. GSH also participates in the nutritional stress response as well as neutralization of harmful substances such as xenobiotics and heavy metals [168]. *M. guilliermondii* possesses two genes encoding GSH: *GSH1* (γ-glutamylcysteine synthetase) and *GSH2* (glutathione synthetase). Inhibition of glutathione biosynthesis results in excessive riboflavin production and elevated iron accumulation within the cells of the yeast *M. guilliermondii.* Under specific conditions in GSH-deficient media, both mutants *ΔGSH1* and *ΔGSH2* display increased riboflavin synthesis (365- and 148-fold, respectively) [169].

The intricate interplay between glutathione biosynthesis and riboflavin production highlights how specific genetic factors and regulatory mechanisms shape metabolic outcomes in *M. guilliermondii*, providing a foundation for further exploration of key regulators. SEF1, a transcription factor in *C. famata,* plays a crucial role in iron-dependent regulation of riboflavin biosynthesis. Deletion of the *SEF1* homologue in *M. guilliermondii* abolished oversynthesis of riboflavin, even under iron-deficient conditions. Conversely, inactivation of *TUP1,* a global repressor in *Candida albicans,* led to increased riboflavin production and iron accumulation in *M. guilliermondii* [170].

Another important aspect of riboflavin metabolism in *M. guilliermondii* involves the transport of riboflavin into and out of the cell. Two key proteins are involved in this process: riboflavin permease [158,171], which facilitates the uptake of riboflavin into the cell, and riboflavin excretase, which is responsible for exporting riboflavin outside the cell [172]. Mutants that are defective in riboflavin excretase show a significant accumulation of riboflavin within the cells, as they are unable to efficiently transport it out. When these excretase-deficient mutants are combined with the overexpression of regulatory genes involved in riboflavin biosynthesis, the result is a dramatic increase in riboflavin accumulation. In fact, the riboflavin levels in these modified yeast cells can reach up to 1000-fold higher than the normal riboflavin content, significantly enhancing riboflavin production [157]. This strategy highlights the potential of manipulating both metabolic regulation and transport mechanisms to optimize riboflavin production in industrial applications.

## 7. *Candida famata* (*flareri*)

*C. famata* is a non-conventional yeast capable of overproducing riboflavin. In 1985, it was classified as an anamorph of *Debaromyces hansenii*, until a new proposal of its teleomorph, *Debaryomyces subglobosus,* appeared in 2008 [173] (Figure 4). A characteristic phenotype of *C. famata* is its high salt tolerance (up to 2.5M NaCl) [174]. Although the complete genome of *C. famata* has not been published, multiple draft assemblies of its teleomorph, *D. subglobosus*, exist [175,176]. Sequenced fragments of *RIB* genes (more specifically amino acid sequences) from *C. famata* show high homology to the *D. hansenii CB767* strain [30]. Therefore, *D. hansenii* genes are frequently utilized for genetic modifications in *C. famata*.

The first strain used commercially for industrial purposes was *C. famata* dep8 (ATCC 20849), which is able to produce over 20 g/L riboflavin in 200 h of cultivation [177], but because of genetic instability it was abandoned for industrial use [14]. The selection method for *C. famata* dep8 was based on cultivation on iron-rich or nutrient-deficient media after mutagenesis using chemicals (deoxyglucose or tubercidin) or radiation and selecting mutants able to grow under limiting conditions [177].

While the *C. famata* dep8 strain was initially developed for industrial riboflavin production, further research identified the key role of the *SEF1* gene in regulating riboflavin synthesis, leading to the development of genetically stable strains with improved riboflavin yields. The *SEF1* gene belongs to a group of regulatory genes responsible for transcription activation. In 2006, a new function was assigned to the *SEF1* gene as a regulatory gene for riboflavin synthesis [178]. This function was clarified due to observation that deletion of the *SEF1* gene led to inability to overproduce riboflavin in *C. famata* [66,178].

In the pathogenic flavinogenic yeast *Candida albicans*, the GATA-type transcription factor Sfu1 is known to negatively regulate *SEF1* expression. In *C. famata*, deletion of the *SFU1* gene in a wild-type strain similarly triggered riboflavin overproduction, suggesting a potential role in flavinogenesis regulation. Furthermore, disruption of the *VMA1* gene, which encodes the vacuolar ATPase subunit A, also led to enhanced riboflavin synthesis in *C. famata* [66].

In 2011, the wild-type strain of *C. famata*—VKM Y-9—was used for classic mutagenesis. Using several chemicals in six stages, a riboflavin-overproducing strain, AF-4, was isolated, which accumulated nearly 680 mg riboflavin/L. Unlike dep8, this strain was genetically stable. The same strain, AF-4, was further improved by inserting an additional copy of *SEF1* (a putative transcription factor from *D. hansenii*), *IMH3* from *D. hansenii* encoding IMP dehydrogenase, and two genes from the first and last steps of riboflavin synthesis: *RIB1* (GTP cyclohydrolase II) and *RIB7* (riboflavin synthetase), respectively. This strain, named BRP (Best Riboflavin Producer), is capable of producing 1 g riboflavin/L in flask culture [179] and up to 16.4 g/L in optimized medium during fed-batch cultivation [46].

As outlined earlier, PRPP is a key metabolite in the de novo purine nucleotide synthesis pathway, where it contributes to the formation of IMP, the central precursor for both AMP and GMP. GMP can then be converted into GTP, which plays a crucial role in riboflavin biosynthesis. Although PRPP is essential for purine metabolism, its involvement in riboflavin synthesis is indirect, acting through its role in nucleotide biosynthesis. Two genes involved in this process, *PRS3* (PRPP synthetase) and *ADE4* (PRPP amidotransferase), were cloned into *C. famata* BRP strain. Both genes were modified to prevent enzyme feedback inhibition by purine pathway products (ADP, GTP). Specific amino acid substitutions were performed to eliminate inhibition: leucine located at positions 132 and histidine 195 in *PRS3* (ADP inhibition) were replaced with isoleucine and glutamine, while in *ADE4* (ATP and GTP inhibition), aspartic acid 315, lysine 338, and alanine 422 were replaced with valine, glutamine, and tryptophan, respectively. The recombinant strain BRPI (**B**est **R**iboflavin **P**roducer **I**mproved) demonstrated significant advancements in riboflavin biosynthesis, with a notable two-fold increase in riboflavin production and enhanced accumulation of guanosine triphosphate (GTP) [68]. Building on this progress, additional genetic modifications were introduced to further enhance the strain’s productivity. Specifically, the overexpression of *RIB1* and *RIB6*, genes coding for GTP cyclohydrolase II and DHBP synthase, respectively, both critical in the riboflavin biosynthetic pathway, was achieved through cloning into the *C. famata* BRPI strain. This strategic enhancement led to a 1.3-fold increase in riboflavin yield, reaching 849 mg/L, compared to the parental strain’s yield of 653 mg/L [36].

The second precursor involved in riboflavin synthesis is Ru5P, which originates from the PPP. Among the genes involved in Ru5P synthesis, *ZWF1*, *SOL*, and *GND1* encode enzymes responsible for key reactions: glucose-6-phosphate dehydrogenase (G6PD), 6-phosphogluconolactonase, and 6-phosphogluconate dehydrogenase, respectively. The name of the *ZWF* gene originates from *Zwischenferment,* meaning ‘intermediate enzyme’. The *ZWF1* gene product catalyzes the first reaction of PPP when glucose-6-phosphate (G-6-P) converts into 6-phosphoglucono lactone (PGL) with simultaneous NADPH production. PGL undergoes another conversion in the presence of 6-phosphoglucono lactonase, the product of the *SOL* gene. The final stage involves participation of 6-phosphogluconate dehydrogenase, the product of *GND1,* in the decarboxylation of glucose, resulting in Ru5P synthesis and the production of a second NADPH molecule [180]. An increased level of Ru5P enhances riboflavin synthesis, as demonstrated by the overexpression of the *GND1* gene under the control of the *TEF1* promoter. This modification led to a 1.3–2-fold increase in riboflavin synthesis compared to the parental strain upon cultivation on whey with addition of ammonium sulfate [181].

It was found that *C. famata* can use lactose as its sole carbon and energy source and exhibits β-galactosidase activity [182]. Overexpression of the *SEF1* transcription activator under the regulation of the lactose-inducible promoter *LAC4* in non-reverting mutant strain *C. famata* AF-4 resulted in a more than two-fold increase in riboflavin yield on lactose compared to the parental strain, AF-4. Moreover, riboflavin synthesis on whey reached 1.69 g/L in shake flasks [183]. The addition of ammonium sulfate to the whey boosted riboflavin production nearly four-fold. Overexpression of the *RIB6* gene in the *C. famata* BRPI strain resulted in substantial enhancement of riboflavin yield on whey, reaching 2.5 g/L in bioreactor cultivation [181].

Further investigations into riboflavin secretion mechanisms have provided additional insights into improving yield. Tsyrulnyk et al. found similarity between the *BCRP* gene (protein of mammary glands responsible for riboflavin secretion into milk) and riboflavin excretase (*RFE1*) from *D. hansenii*. Both of these are responsible for excretion of riboflavin outside the cell. Overexpression of the *D. hansenii RFE1* gene into the *C. famata* BRP strain resulted in a higher riboflavin yield, reaching up to a 1.8-fold higher level compared to the parental strain. This modification led to a significant reduction in the intracellular riboflavin pool. Interestingly, the modified strain (*C. famata BRP/RFE1*) also exhibited increased expression of two other genes, *RIB1* and *RIB6*, compared to the parental strain, suggesting a direct impact on the regulation of these *RIB* genes [184].

To further enhance riboflavin utilization and its biotechnological applications, it is crucial to understand its conversion into active cofactors. Due to their higher water solubility and greater therapeutic efficacy, these flavin nucleotides are often preferred over riboflavin in pharmaceutical and food industry applications. In eukaryotic cells, riboflavin is transformed into the catalytically active cofactors FMN and FAD through a sequential process (Figure 1). This process involves riboflavin phosphotransferase (riboflavin kinase), which phosphorylates riboflavin to produce FMN, followed by FMN adenylyltransferase (FAD synthetase), which converts FMN in the presence of ATP into FAD by adenylation [14,131,185].

As mentioned before, eukaryotes possess separate genes for riboflavin kinase and FAD synthetase, namely *FMN1* and *FAD1*, respectively [185]. Overexpression of the *FMN1* gene under a strong *TEF1* promoter results in a 400-fold increase in FMN production in a recombinant *C. famata* strain (318.2 mg/L) [131].

FAD can be isolated from *E. ashbyii*, but a recombinant *C. famata* strain has been reported to accumulate FAD synthesized de novo. Yatsyshyn et al. overexpressed the *FAD1* gene from *D. hansenii* under the *TEF1* promoter in a *C. famata* mutant strain containing an additional *FMN1* gene under the same strong promoter [58]. This led to the production of 451.5 mg/L of FAD after 40 h of batch cultivation in a bioreactor on modified Burkholder medium.

Beyond its role in cofactor biosynthesis, *C. famata* has also attracted interest for its potential in synthesizing riboflavin-derived bioactive compounds. In addition to producing riboflavin, *C. famata* is considered a promising organism for the production of antibacterial drugs—roseoflavin and aminoriboflavin. Roseoflavin and its biosynthetic precursor, 8-aminoriboflavin, are natural riboflavin analogs and antibiotics produced in minimal quantities by certain bacteria, such as *Streptomyces davaonensis*. Genes involved in aminoriboflavin synthesis (*rosB*, *rosC*) have been effectively implemented in a *C. famata* mutant strain overproducing FMN (immediate antibiotic precursor). Although the *rosA* gene was also expressed, isolating a roseoflavin-producing strain of *C. famata* was unsuccessful due to a lack of activity of the *RosA* enzyme. All three genes were successfully expressed in the riboflavin-overproducing mutant of another non-conventional yeast, *Komagataella phaffii (Pichia pastoris)*. These antibiotics are effective antibacterial agents, making their heterologous overproduction in yeasts a promising avenue for medical implementation [186,187].

## 8. Comparison of Microbial Riboflavin Producers (Advantages and Disadvantages)

The production of riboflavin (vitamin B2) through microbial fermentation is an important biotechnological process, and several microorganisms have been explored for industrial riboflavin production. Among these, *Candida famata*, *Bacillus subtilis*, and *Ashbya gossypii* are the most extensively studied species [10].

*C. famata* is a well-known riboflavin overproducer that was previously used for large-scale industrial production. The production efficiency of riboflavin from this yeast can reach high levels (more than 20 g/L), depending on the optimization of fermentation conditions. However, due to its genetic instability, its use for industrial riboflavin production was terminated [14]. *A. gossypii* became the preferred eukaryotic riboflavin producer, achieving over 20 g/L of riboflavin [5,14]. By comparison, the yield of riboflavin in *B. subtilis* has reached nearly 35 g/L [188].

When it comes to fermentation substrates, *C. famata* typically grows well on carbohydrates such as glucose, lactose, galactose, sucrose, maltose, trehalose, D-xylose, L-arabinose, melezitose, glycerol, raffinose, cellobiose, D-mannitol, D-glucitol, ribitol, salicin, DL-lactic acid, and succinic acid [174] or industrial wastes such as whey [181] and lignocellulose hydrolysate [189]. The fungus *A. gossypii* can ferment a wide variety of substrates, including glucose, sucrose, fructose, starch, maltose, and glycerol, which promote good growth, and even agro-industrial residues such as crude glycerol, whey, corn oil, and soybean oil, thereby promoting riboflavin synthesis. This versatility is a significant advantage in terms of sustainability and cost-effectiveness [133]. In comparison, *B. subtilis* benefits from utilizing basic raw materials, such as glucose, sucrose, starch, or glycerol [190].

Another key factor is time, as riboflavin production can occur at different growth stages. Among the organisms studied, *B. subtilis* demonstrates the fastest growth rate. However, its growth-associated riboflavin production poses the challenge of potentially favoring the emergence of non-producing mutants during cultivation [10]. *C. famata*, despite slower growth, produces riboflavin from the onset of the trophophase—exponential growth phase. In *A. gossypii,* riboflavin synthesis begins only when growth reaches the stationary phase [133]. This delay in productive growth can be perceived as a disadvantage.

In most organisms, the codon CUG typically codes for the amino acid leucine. However, some exceptions exist, where CUG is decoded differently, a phenomenon known as alternative codon usage (Figure 4). For example, in some species of the genus *Candida*, including *C. albicans* and *C. famata*, the codon CUG is translated as serine instead of leucine. This alternative usage is facilitated by a modified tRNA with a unique anticodon structure and aminoacylation machinery [191,192]. While this alternative codon usage can offer an advantage in adapting to unfavorable environmental conditions, it may also pose a limitation. Substituting leucine residues with serine residues can disrupt the protein’s three-dimensional structure or change its surface properties due to the change in hydrophobicity, which may impact its activity and/or stability [193]. In certain mitochondrial genomes, codon reassignment has been also observed. Mitochondria from *A. gossypii*, instead of leucine, decode alanine using CUA and CUU codons [194].

The differences between *C. famata, A. gossypii,* and *B. subtilis* are summarized in Table 2.

## 9. Riboflavin Production from Waste Products

Modern fermentation technologies have significantly advanced industrial bioprocessing, enhancing efficiency and scalability. Bioreactors, which are controlled environments for growing microorganisms, play a crucial role across scientific research and industrial applications. Incorporating diverse sensors is vital for precise real-time monitoring, early detection of issues, ensuring reproducibility, minimizing costs, and improving overall efficiency [203]. The success of a bioprocess relies on a seamless connection between feedstock selection and process design. By utilizing sustainable feedstocks and tailoring bioprocess parameters, industries can reduce costs, enhance yields, and promote environmentally friendly production practices.

Agricultural waste, such as lignocellulosic biomass, comes from residues like straw, corn stover, sunflower stalks, and sugarcane bagasse. Depending on the type of residue, pretreatment and hydrolysis methods are applied, resulting in varying sugar compositions and inhibitor contents and concentrations. Pentoses such as xylose and L-arabinose are often inefficiently metabolized by microorganisms. However, as mentioned earlier, *C. famata* is particularly well-suited for riboflavin production from sugar mixtures due to its ability to grow on diverse substrates, including xylose and L-arabinose. The most efficient strains capable of synthesizing riboflavin from such residues may be further enhanced through targeted strategies and genetic manipulations aimed at optimizing both sugar utilization and resistance to hydrolysate inhibitors, ultimately improving overall process efficiency.

Building on the efforts to enhance riboflavin secretion and optimize substrate utilization, recent research has explored alternative feedstocks for *C. famata* fermentation. A recent study demonstrated that *C. famata* strains can also be used to produce riboflavin from waste lignocellulosic hydrolysates, which are rich in various sugars, mainly glucose, xylose, and L-arabinose. To further improve the efficiency of this process, the focus was on better xylose utilization, which would translate into better production. For this purpose, additional copies of the *XYL1* and *XYL2* genes (xylose reductase and xylitol dehydrogenase, respectively) were introduced into the BRPI strain. The engineered strain exhibited increased riboflavin production, which reached 1.5 g/L during a bioreactor using bagasse hydrolysate as the carbon source [189].

While classical lignocellulosic biomass is primarily composed of cellulose, hemicellulose, and lignin, certain plant-derived materials also contain significant amounts of pectin. Hydrolysates derived from such biomass are often rich in L-arabinose, with beet pulp being a notable example. In addition to proteins, lipids, and minerals, beet pulp contains various component sugars, which constitute 68% of its dry matter. Among them, D-galacturonic acid and L-arabinose are the two dominant carbon compounds, accounting for more than 70% and 18% of the dry matter, respectively [204].

Previous studies have focused mainly on the optimization of xylose metabolism, as its efficient conversion has been a significant challenge for maximizing riboflavin production in *C. famata*. Although this strain has demonstrated satisfactory growth results on L-arabinose as indicated in previous studies, our observations suggest that further enhancement of its utilization could significantly improve the entire fermentation process in the case of hydrolysates rich in this pentose.

Despite the mentioned modifications aimed at improving growth on pentoses, a more critical challenge is that *C. famata* exhibits relatively low resistance to inhibitors present in lignocellulosic hydrolysates, which are generated during the processing of lignocellulosic biomass. Notably, efficient production occurs only on diluted hydrolysates. Compounds such as acetic acid, 5-hydroxymethylfurfural (HMF), and furfural can induce oxidative stress in cells, leading to damage to key biomolecules, disruption of cellular energy metabolism, membrane damage, inhibition of essential metabolic enzymes, impaired nutrient transport, DNA damage, and the accumulation of toxic metabolites. Consequently, these effects may result in a substantial decline in production efficiency [205,206]. Further studies are ongoing to enhance the resistance of *C. famata* to inhibitors present in hydrolysates (unpublished data).

Another type of agricultural waste is the solid waste discharged by an oil refinery plant that contains waste rapeseed or palm oil. *A. gossypii* can utilize a variety of vegetable oils wastes to produce riboflavin [207,208,209].

Different groups of waste come from the food industry. Whey is a by-product of the cheese-making process and is rich in lactose, which can serve as a carbon source for a minority of microorganisms. The composition of nutrients varies depending on the type of whey used (sweet or acid) [210] as well as proteins and lipid content. *C. famata* strains can metabolize the lactose in whey to produce riboflavin during fermentation. Addition of ammonium sulfate boosts production even more [181]. Furthermore, cultivation of *A. gossypii* in whey with the addition of soybean flour yielded better results in riboflavin production than supplementation with yeast extract, glycine, or sucrose [211]. Besides dairy waste, fruit and vegetable peels represent another category of food industry waste. A mutant strain of *Ashbya gossypii* has been reported to utilize 0.3% orange rind in YM medium, leading to a 184% enhancement in riboflavin production. [212]. Citrus molasses come from water pressed through waste peel, rag, seeds, and residual juice. It can be utilized as a raw material for the fermentation process by *A. gossypii* NRRL 1363 to produce riboflavin [213]. Similarly, molasses (dextrose substitutes) with the addition of peanut seed cake (nitrogen source) and other nutrients is another option for riboflavin production using *E. ashbyii* [214].

Brewer’s spent grain (BSG) is a major by-product of beer-brewing. Composed of protein and fiber such as cellulose, arabinoxylan, and lignin, BSG is classified as a lignocellulosic material. It has been reported that it also contains vitamins, amino acids, minerals, and oligo- and polysaccharides. BSG requires pre-treatment to become accessible to microorganisms. Although use of BSG as media for the microbial production of enzymes, xylitol, citric acid, and others has been reported [215], there is no information about riboflavin production using this type of waste by-product. However, it has been reported that, under specific conditions and supplementation, *Candida guilliermondii* (strain ATCC 9058) is able to produce riboflavin from liquid brewery waste [216].

Industrial crude glycerol, a by-product of biodiesel production, has been used as a substrate for various microbial processes. *A. gossypii* has been reported to produce orotic acid from crude glycerol; however, there is no available information on riboflavin yield from this type of renewable feedstock [217]. Riboflavin synthesis in *C. famata* during cultivation on crude glycerol was not studied.

## 10. Conclusions and Prospects

This review compares the genetic regulatory mechanisms of prokaryotes and eukaryotes involved in riboflavin production. Emphasis is placed on effective application strategies for microorganisms such as *B. subtilis*, *A. gossypii*, *C. famata*, and *M. guilliermondii* in riboflavin overproduction, including genetic manipulation, metabolic pathway regulation, and culture condition optimization., etc. Additionally, the characteristics of different microorganisms are compared, analyzing their advantages and disadvantages in riboflavin production to provide a theoretical basis for optimizing production systems. Significant attention was also paid to the biotechnological production of flavin nucleotides (FMN, FAD) and flavin antibiotics (roseoflavin, aminoriboflavin), which hold great potential for implementation in medical practice, and also, for FMN, in the food industry.

Reducing costs, increasing riboflavin yield and productivity, and efficiently utilizing resources will remain key research priorities in the future. In particular, the use of industrial waste as substrates for riboflavin production is an inevitable trend, aligning with the principles of sustainable and green manufacturing. With advancements in synthetic biology and metabolic engineering, further exploration and optimization of riboflavin-overproducing strains will promote industrial riboflavin production toward greater efficiency, sustainability, and economic feasibility.

## Figures and Tables

**Table 1:** Key differences between chemical synthesis and fermentation in riboflavin production. (Adapted from [5,7,8]).

Aspect	Chemical Synthesis	Fermentation	Reference(s)
Process Overview	Uses multiple chemical reactions to produce riboflavin from simpler chemical precursors.	Employs microorganisms (e.g., *A. gossypii* or *B. subtilis*) to biologically produce riboflavin in single-step fermentation.	[5,7]
Raw Materials	Requires D-ribose or D-glucose.	Requires substrates such as glucose or waste by-products e.g., corn steep liquor or vegetable oils.	[5,7]
Energy Requirements	The process is highly energy-intensive because of multiple reaction steps, high temperatures, and high pressures.	Relatively low energy requirements; operates under milder conditions (normal pressure, mild temperature).	[3,9]
Environmental Impact	Generates chemical waste and potentially harmful by-products.	Environmentally friendly; low waste generation and biodegradable by-products.	[6,7]
Yield and Efficiency	Moderate yield; dependent on reaction optimization and catalyst efficiency.	High yield; microorganisms can be genetically modified to increase efficiency.	[10,11]
Cost	High cost as a result of energy consumption and raw materials.	Lower long-term cost, particularly with optimized fermentation processes.	[10,11]
Quality Control	Consistent product quality due to controlled chemical reactions.	Product quality can vary depending on the microorganism used and fermentation conditions.	[9,10]
Time Frame	Typically shorter time frame as reactions occur quickly.	Longer process, as it depends on microbial growth and metabolism.	[7]
Sustainability	Less sustainable as a result of reliance on fossil-derived inputs and high energy use.	Sustainable, especially when using renewable feedstocks.	[7,9]
Applications	Used when rapid production is needed or for industrial processes that can accommodate high costs.	Preferred for large-scale production, especially in the food, feed, and pharmaceutical industries.	[3,6]

**Table 2:** Characteristics and applications of *C. famata*, *A. gossypii*, and *B. subtilis*.

Feature	*Candida famata*	*Ashbya gossypii*	*Bacillus subtilis*	Reference(s)
Morphology	Yeast-like; unicellular	Filamentous fungus; forms mycelium	Gram-positive bacterium; rod-shaped	[102,174,195]
Habitat	Found in all types of cheese, in dairies, and in brine	Found in plants like cotton	Found in soil (especially near plant roots—rhizosphere)	[133,174,196]
Ecological Role	Environmental and commensal yeast	Plant pathogen	Decomposer, plant-growth promoter, and biocontrol agent	[196,197,198]
Genome size	*D. hansenii* 12.2 Mb *D. subglobosus* 11.5 Mb	9.2 Mb	~4.2 Mbp	[140,176,199,200]
Riboflavin yield	Over 20 g/L	Over 20 g/L	Over 34 g/L	[5,177,188]
Substrate versatility	Wide range of substrates	Prefers inexpensive, plant-based oils	Utilizes a variety of carbohydrates, like glucose and sucrose	[133,174,200]
Genetic engineering	Effective but less developed tools, alternative CUG coding	Advanced tools for metabolic engineering, e.g., CRISPR/Cas9	Overexpression of the *rib* operon; disruption of feedback inhibition of FMN riboswitch	[11,133,151,174,200]
Industrial use	Industrial use was terminated due to genetic instability	Primary organism for riboflavin production	Widely used in industrial-scale production of riboflavin	[3,133,201]
Osmotic and saline tolerance	Osmotolerant and halotolerant	Grows best under moderate osmotic and salt conditions	Moderate tolerance to osmotic and saline stress	[133,174,202]
Riboflavin synthesis phase	Production starts in growth phase	Production starts in stationary phase	Production starts in the late exponential to early stationary phase of growth	[6,133]
